# Effects of dietary clove oil on growth performance, blood biochemistry and lipid metabolism in broiler chickens fed low-energy diets

**DOI:** 10.1038/s41598-025-27183-7

**Published:** 2025-12-08

**Authors:** Niamat M. El-Abd, Ragaa A. Hamouda, Marwa Salah Abdel-Hamid

**Affiliations:** 1 Department of Sustainable Development of Environment, Environmental Studies and Research Institute, University of Sadat City, 22857 Sadat City, Menoufyia Governorate Egypt; 2 Department of Applied Radiologic Technology, College of Applied Medical Sciences, University of Jeddah, Jeddah City, 23218 Saudi Arabia; 3 Microbial Biotechnology Department, Genetic Engineering and Biotechnology Research Institute, University of Sadat City, 22857 Sadat City, Menoufyia Governorate Egypt

**Keywords:** Broiler chickens, Emulsified clove oil, Growth performance, Oxidative stress, Fatty acid, Abdominal microbial populations, Microbiology, Zoology, Health care

## Abstract

This study investigated the effects of emulsified clove oil supplementation on growth performance, carcass characteristics, blood biochemical parameters, antioxidant status, and meat quality of broiler chickens fed a low-energy diet. A total of 252 day-old Cobb broiler chicks unsexed were randomly assigned to three groups: a control group, T1 (300 mg/kg clove oil with emulsifier and 200 kcal energy reductions), and T2 (300 mg/kg clove oil without emulsifier and 200 kcal energy reduction). Both treatments improved body weight gain and feed conversion ratio, with T1 showing superior efficiency due to enhanced nutrient absorption. Carcass analysis revealed increased yields of breast and thigh, especially in T2, while T1 supported more balanced growth. Blood biochemical results indicated improved protein metabolism and lipid profile without adverse effects on liver or kidney function. The antioxidant capacity was significantly elevated in both treated groups, resulting in a reduction of oxidative stress markers. Clove oil supplementation also altered the fatty acid profile of broiler meat by increasing monounsaturated fatty acids and maintaining polyunsaturated fatty acid levels. Notably, the emulsifier in T1 significantly reduced abdominal microflora by up to 55% compared to other groups. MALDI-TOF/MS Biotyper^®^ identified the dominant microorganisms as *Bacillus altitudinis*/pumilus. These findings highlight the potential of emulsified clove oil as a functional feed additive for enhancing broiler productivity and health.

## Introduction

The poultry industry has increasingly focused on natural feed additives to enhance growth performance, nutrient utilization, and overall health in broilers. Essential oils, particularly clove oil, have gained attention due to their antimicrobial, antioxidant, and digestive-enhancing properties. Clove oil, derived from *Syzygium aromaticum*, is rich in bioactive compounds such as Eugenol, which has been reported to improve feed efficiency and immune responses in poultry^1^. One of the major challenges in broiler production is optimizing feed conversion efficiency while maintaining gut health and immune function. Antibiotic growth promoters (AGPs) were traditionally used to enhance these aspects, but due to growing concerns over antibiotic resistance, alternative strategies are being explored^2^. Phytogenic additives, including essential oils, have emerged as viable substitutes due to their ability to modulate intestinal microbiota and improve digestion^2^.

Studies have shown that clove oil supplementation positively influences broiler performance by enhancing nutrient absorption, reducing oxidative stress, and improving immune responses^4^. However, the effectiveness of clove oil can be influenced by its dosage and formulation. Emulsification is one method that has been suggested to improve the bioavailability of essential oils, potentially enhancing their benefits^5^. Due to antibiotic residues in finished meat products and the emergence of antibiotic-resistant bacterial strains in consumers, there has been an increasing call since 2006 to phase out the antibiotics used as growth promoters in poultry production. Consumer opposition to synthetic chemicals has been growing recently. Digestive enzyme activity is increased by these oils, which have been demonstrated to have a good impact on a number of metabolic processes, including lipid metabolism^6^, and provide anti-inflammatory, antibacterial, antioxidant, and immunomodulatory effects, hence enhancing gut health and broiler growth performance overall. Consequently, in broiler nutrition, essential oils are being explored as possible antibiotic substitutes; the efficacy of each essential Oils varies according to its unique bioactive constituents. The primary objective of this study is to assess the effects of clove essential oil (*Syzygium aromaticum*), which is abundant in humulene, eugenol, caryophyllene, and humulene epoxide, on the growth and health of broilers. These elements are thought to be essential for enhancing animal performance and health. The antibacterial, antifungal, and antioxidant properties of clove essential oil have been documented^7^, these properties make clove essential oil a viable substitute for antibiotics in broiler diets, as it can enhance growth, immune system performance, and gut health. Clove essential oil is rich in phenolic compounds, which have a variety of biological actions, such as insecticidal, antifungal, antibacterial, and antioxidant qualities^8^.

Studies have shown that clove oil supplementation positively influences broiler performance by enhancing nutrient absorption, reducing oxidative stress, and improving immune responses^9^. However, the effectiveness of clove oil can be influenced by its dosage and formulation. The addition of an emulsifier is one method that has been suggested to improve the bioavailability of essential oils, potentially enhancing their benefits. Emulsifiers help in dispersing the oil more effectively within the feed, making it more accessible to the digestive system^10^. Therefore, this research investigates the effects of clove oil and emulsifier supplementation on broiler chicks performance and health parameters and aims to evaluate the effects of clove oil treatment, with and without emulsifier, on broiler performance, carcass characteristics, blood parameters, antioxidant status, and microbial content.

## Materials and methods

### Ethical approval

The experiment was conducted at the United Farm, Kafr El Sheikh City, Egypt. under licence number SREC30425B20060 for animal experimentation, in compliance with the Genetic Engineering and Biotechnology Research Institute, University of Sadat City, Egypt, and was approved by the local ethics committee for animal experimentation. The study followed the ARRIVE guidelines.

### Experimental design

This trial was conducted at a private poultry farm for duration of 35 days. A total of 252 day-old Cobb broiler chicks unsexed were randomly assigned to three experimental groups, with each group consisting of three replicates of 28 chicks per replicate.

The dietary treatments were formulated as follows: Control: A basal diet with no supplementation (T1). Basal diet with a 200 kcal energy reduction supplemented with 300 mg clove oil emulsifier by 1 g emulsifier and adds to kg diet (T2). Basal diet with a 200 kcal energy reduction, supplemented with 300 mg clove oil/kg diet. The 300 mg clove oil used in this study was obtained from a verified supplier to ensure quality and purity. The emulsifier, composed of phospholipids, glycolipids, and triglycerides, was sourced from a commercial manufacturer specializing in poultry feed additives. All chicks were raised under standard environmental and management conditions, with unrestricted access to feed and water. The experimental diets were formulated to evaluate the effects of energy reduction on broiler performance. Treatments T1 and T2 were designed with a reduction of 200 kcal/kg in metabolizable energy compared to the control diet. Despite the reduction in energy, the diets were adjusted to maintain comparable levels of crude protein, amino acids (such as lysine and methionine), and other essential nutrients across all groups. Thus, the diets were isonitrogenous but not isocaloric, as the aim was to investigate the influence of energy dilution while maintaining overall nutritional balance. All diets were formulated in Table 1 according to the nutrient requirements of broilers as recommended by NRC^11^ and adapted to the experimental design.

The experimental design followed a completely randomized design (CRD), ensuring uniform allocation of treatments. Throughout the 35-day experimental period, feed and water were provided ad libitum. All experimental groups were maintained under identical lighting and temperature conditions. A continuous light schedule was applied, with 24 h of light from day 0 to day 7, followed by 23 h of light per day from day 8 until the end of the trial. The ambient temperature in the rearing room ranged from 25 °C to 29 °C, with relative humidity maintained between 50% and 70%. Body weight of the birds was recorded every three days, while daily feed intake was monitored and recorded throughout the entire trial period.

### Measurements and methods

Live Body weight (LBW), feed intake (FI), feed conversion ratio (FCR), and body weight gain (BWG) were the parameters that were computed and/or determined for the feeding trial. Crude Protein Utilization (%) and Ether Extract Utilization **(%)** were calculated to assess the efficiency with which birds converted dietary crude protein and ether extract into body mass. The following formulas were used:

Crude Protein Utilization (%) = (Protein intake – Protein in excreta)/Protein intake × 100.

Ether Extract Utilization (%) = (Ether extract intake – Ether extract in excreta)/Ether extract intake × 100.

Following the trial’s at 35 days, as soon as the birds were slaughtered. Sterile tubes were used to collect and store blood samples. The samples were centrifuged at 3000 rpm for 10 min to separate the serum. The serum was then carefully collected and stored at − 20 °C until further biochemical analysis. Using a spectrophotometer, the following parameters were measured on an individual basis: cholesterol (CHO) (mg/dL), aspartate aminotransferase (AST) (u/L), alanine aminotransferase (ALT) (u/L), globulin (GL, g/dL), albumin (Alb, g/dL), and total protein (TP, g/dL). The results were obtained in accordance with the manufacturer’s instructions. Oxidative stress and antioxidant biomarkers, such as MDA, SOD, and catalase (CAT), were assessed.

### Assessing the fatty acid composition of meat

After adding around 2 mL of n-hexane to 0.1 g of tissue and shaking it for 30 min at 50 °C, 3 mL of potassium hydroxide methanol solution (0.4 mol/L) was added. Once at 50 °C and 200 rpm, the samples were agitated for 30 min. Following that, the mixture was blended after the addition of 1 mL of water and 2 mL of n-hexane. In order for stratification, the mixture was left to rest. Gas chromatography (GC)-mass spectrometry was used to identify fatty acids^12^. The chromatographic settings and gas chromatographic equipment (TraceGC model K07332, Hermo Finnigan, Thermo Quest, Milan, Italy) were as follows. N_2_ was used as the carrier, and the oven was run at 70 °C for 0.5 min, 180 °C for 10 min, and 225 °C for 15 min, with increments of 5 °C/min. The detector and intake temperatures were 1 µL with split 1/20 and 250 °C, respectively. Supelco (Milan, Italy) reference standards were used to determine the fatty acid methyl ester (FAME) retention durations and elution order.

### Measurements of the carcass and immunological organs

Forty five birds were randomly selected, (five birds from each replicate) to ensure equal representation across all treatment groups. They were selected at random after 35 days, and their processing yields were assessed. After being denied nutrition for 12 h, the birds were weighed and slaughtered. The head and shoulders were removed, and the bodies were sliced open to separate the breasts and legs. Weighing was done on the fat content, liver, heart, spleen, and thigh. Each piece’s yield % was calculated using the dressing weight as a basis.

### Determination and identification of abdominal macrobiotic contents

The abdominal organs ileum samples (approximately 2 g) were collected immediately after scarified homogenization of all treatments and, serially diluted in 10-fold in phosphate buffered saline. The plate count technique was used to count and enumerate the bacterial community on LB medium (Merck, Germany) incubated at 37 °C for 24 h^13^. The results are presented as (CFU‧mL^− 1^) in triplicate. The results were expressed as the mean ± standard error of log CFU of viable bacteria. Selected colonies were identified at the species level based on matrix‑assisted laser desorption/ionization‑time of flight (MALDI‑TOF) mass spectrometry (MS) biotyper.

The selected isolate was identified via (MALDI-TOF–MS, database version 3, BioMerieux, France). Confidence percentages between 90 and 98% were used for genus-level identification and > 98% for species-level identification and < 90% of the results were considered unacceptable. For identifying the isolate, the peaks from the spectrum were compared to the standard spectrum for a certain species, genus, or family of microbe.

### Statistical analysis

One-way analysis of variance (ANOVA) was used to statistically analyze the results using the Statistical Package for the Social Sciences, version 22 for Windows (SPSS Inc., Chicago IL USA). Duncan’s Multiple Range: To distinguish between significant means at *p* < 0.05, the Duncan’s Multiple Range test was applied^14^. Heatmap correlations of the studied data were visualized by JMP^®^, Version 17.2.0.

**Table 1:** Nutrient composition of broiler Diets.

Ingredient (%)	Control		200 kcal + 300 mg CO + 1gE		200 kcal + 300 mg CO	
	Starter	Grower	Starter	Grower	Starter	Grower
Yellow Corn	55.00	59.00	53.80	57.00	53.30	57.00
Soybean Meal	38.40	33.30	39.00	34.97	39.80	35.47
Soybean Oil	2.38	3.50	1.60	2.70	1.40	2.30
Limestone	1.17	1.35	2.31	2.35	2.31	2.35
Dicalcium Phosphate	2.05	1.78	2.15	1.78	2.15	1.78
Premix*	0.50	0.50	0.50	0.50	0.50	0.50
Salt	0.30	0.30	0.30	0.30	0.30	0.30
DL-Methionine	0.16	0.17	0.17	0.17	0.17	0.17
L-Lysine	0.04	0.10	0.04	0.10	0.04	0.10
Clove Oil (CO)	-	-	0.03	0.03	0.03	0.03
Emulsifier	-	-	0.10	0.10	-	-
Total	100	100	100	100	100	100
Calculated Composition						
Metabolizable Energy (kcal/kg)	3029	3110	2832	2904	2820	2900
Crude Protein (%)	21.91	19.19	22.01	22.00	22.26	20.21

Vitamins and minerals mixture at 0.50% of the diet supplies the following (kg of the diet): vitamin A, 10,000 IU; vitamin D3, 3,000 IU; vitamin E, 24 mg; vitamin K3, 2.1 mg; vitamin B12, 2 mg; riboflavin, 5.0 mg; pantothenic acid, 15 mg; niacin, 40 mg; choline chloride, 500 mg; folic acid, 0.9 mg; vitamin B6, 3.0 mg; biotin, 0.05 mg; Mn, 70 mg; Fe, 80 mg; Zn, 100 mg; Cu, 18.8 mg; I, 0.35 mg; Se, 0.30 mg. Crude protein in nutrient levels were analyzed values, other nutrients were calculated values: DL–methionine: 98% feed grade(98% methionine)according to NRC.

## Results

### Effect of emulsified clove oil supplementation on broiler growth performance

Table 2 summarizes the effects of dietary energy reduction and clove oil supplementation on broiler growth performance, crude protein utilization, and ether extract utilization. Dietary treatments had a significant impact on body weight gain (BWG) and feed conversion ratio (FCR) (*P* < 0.05), while feed intake (FI) showed no significant differences in some weeks (*P* > 0.05). At the end of the trail, T1 achieved the highest live body weight (LBW), followed by T2, while the control group had the lowest values. A similar trend was observed in BWG, with T1 recording the highest gain, followed by T2 and then the control (*P* = 0.001). FI remained comparable across treatments in certain weeks, while FCR was lowest (best) in T1, followed by T2, and highest in the control group. This indicates improved feed efficiency with clove oil, particularly with the emulsifier, crude Protein utilization varied significantly among treatments, with T1 achieving the highest value, followed by T2, while the control had the lowest (*P* = 0.000). This suggests that the combination of clove oil and emulsifier improved protein absorption and metabolism. Similarly, ether extract utilization was highest in T1, followed by T2, with the control group having the lowest value. Both supplemented groups significantly outperformed the control, confirming that clove oil enhanced energy utilization, especially when combined with an emulsifier. T1 demonstrated the best overall performance, with superior LBW, BWG, crude protein utilization, and ether extract utilization, as well as the lowest FCR. Although T2 improved efficiency compared to the control, its performance was slightly lower than T1, likely due to the absence of an emulsifier. These findings indicate that dietary energy reduction with clove oil supplementation enhances feed efficiency, promotes growth, and improves nutrient utilization in broiler chickens (*P* < 0.05).

**Table 2:** Effect of clove oil on growth performance of broiler chicks.

Items	Treatments			
	Control	T1	T2	*P*- value
Initial body weight (g)	70 ± 2^a^	70 ± 1^a^	70 ± 1^a^	0.0.105
Live body weight(g/35 day)	1851.09 ± 4.75^c^	2358.25 ± 7.78^a^	2244.09 ± 12.3^b^	0.000
Body weight gain(g/35 day)	1781.09 ± 2.62^c^	2288.25 ± 5.9^a^	2174.09 ± 9.8^b^	0.001
Feed intake(g/35 day)	3135.78 ± 8.84	3145 ± 17.55	3174.2 ± 11.19	0.108
Feed conversion ratio(g gain/g feed	1.76 ± 0.03^a^	1.37 ± 0.22^c^	1.46 ± 0.38^b^	0.000
Crude Protein utilization%	64 ± 0.02ᶜ	67 ± 0.03ᵃ	66 ± 0.02ᵇ	0.002
Ether extract utilization%	70 ± 0.002ᶜ	76 ± 0.003ᵃ	74 ± 0.002ᵇ	0.003
Mortality number	5	3	3	

Superscript letters (a, b, c) indicate significant differences within rows (*P* < 0.05).

Control: Basal diet with no supplementation, T1: Basal diet with a 200 kcal energy reduction, supplemented with 300 mg clove oil/kg diet and 1 g emulsifier/kg diet, T2: Basal diet with a 200 kcal energy reduction, supplemented with 300 mg clove oil/kg diet.

### Effect of emulsified clove oil supplementation on carcass traits of broiler chicks

The effects of dietary energy reduction and clove oil supplementation on carcass traits and lymphoid organ weights in broiler chickens are summarized in Table 3. The final body weight was significantly influenced by dietary treatments (*P* = 0.004), with T1 achieving the highest weight, followed by T2, while the control group had the lowest. Although carcass weight did not differ significantly (*P* = 0.304), the breast muscle percentage was notably higher in T1 and T2 compared to the control (*P* = 0.004), suggesting improved lean meat yield. The thigh muscle percentage was significantly greater in T1 and T2 than in the control (*P* = 0.011), indicating enhanced muscle growth. Meanwhile, abdominal fat percentage remained unaffected (*P* = 0.119), suggesting that dietary modifications did not impact fat deposition. However, liver weight% was higher in supplemented groups (*P* = 0.049), possibly due to increased metabolic activity. The spleen weight was significantly greater in T2, with T1 showing a similar trend (*P* = 0.041). Additionally, the thymus weight was higher in supplemented groups (*P* = 0.001), reflecting enhanced immune function. A similar pattern was observed for the Bursa of Fabricius (*P* = 0.005), further supporting improved immunity. T1 demonstrated the best overall performance, showing higher body weight, lean muscle yield, and improved immune system development. While T2 also enhanced performance, it was slightly less effective, likely due to the absence of an emulsifier. The control group consistently exhibited the lowest values, confirming that clove oil supplementation, particularly with an emulsifier, enhances growth, carcass traits, and immune function in broilers (*P* < 0.05).

**Table 3:** Effect of clove oil on carcass traits of broiler chicks.

Items	Treatments			*P*
	Control	T1	T2	
Body weight	1950 ± 28.86^c^	2150 ± 28.86^a^	2080 ± 15.27^b^	0.004
Carcass weight	0.75 ± 0.005^a^	0.76 ± 0.008^a^	0.76 ± 0.005^a^	0.304
Breast muscle %	23.3 ± 0.33^b^	25.3 ± 0.33^a^	26 ± 0.57^a^	0.004
Thigh muscle %	9.9 ± 0.120^b^	10.3 ± 0.08^a^	10.2 ± 0.14^a^	0.011
Abdominal %	2.5 ± 0.03^a^	2.76 ± 0.08^a^	2.7 ± 0.05^a^	0.119
Liver%	1.83 ± 0.03^a^	1.9 ± 0.005^a^	1.91 ± 0.005^a^	0.049
Lemphiod organs				
Spleen mg/g BW	1.24 ± 0.003^b^	1.30 ± 0.005^b^	1.27 ± 0.01^a^	0.041
Thymus mg/g BW	2.14 ± 0.005^b^	2.21 ± 0.005^a^	2.19 ± 0.00^a^	0.001
Bursa Fabricius mg/g BW	1.20 ± 0.003^b^	1.25 ± 0.003^a^	1.23 ± 0.003^a^	0.005

Superscript letters (a, b, c) indicate significant differences within rows (*P* < 0.05).

Control: Basal diet with no supplementation, T1: Basal diet with a 200 kcal energy reduction, supplemented with 300 mg clove oil/kg diet and 1 g emulsifier/kg diet, T2: Basal diet with a 200 kcal energy reduction, supplemented with 300 mg clove oil/kg diet.

### Impact of energy reduction and clove oil supplementation

Reducing dietary energy with clove oil supplementation significantly influenced blood parameters, antioxidant status, and oxidative stress markers in broilers (Table 4). In terms of total protein (TP), T2 recorded the highest values, whereas T1 showed the lowest (*P* = 0.008). This suggests that while clove oil supplementation can enhance protein metabolism, the presence of an emulsifier in T1 may have affected protein utilization. Similarly, albumin (Alb) levels were significantly lower in T1 and T2 (*P* = 0.002), indicating potential shifts in protein metabolism due to dietary modifications. Moreover, cholesterol (CH) was significantly higher in the clove oil groups (*P* = 0.031), possibly due to improved fat digestion and absorption. However, creatinine (Creat) levels remained unchanged (*P* = 0.416), confirming that dietary treatments did not impair kidney function. Additionally, liver enzymes (AST, ALT) were lower in T1, suggesting improved liver health and metabolic efficiency. Notably, total antioxidant capacity (TAC) increased significantly in T1 and T2 (*P* = 0.001), demonstrating that clove oil effectively enhances antioxidant defense. Furthermore, malondialdehyde (MDA), an oxidative stress marker, was lowest in T2 (*P* = 0.000), which implies that a higher clove oil dose provides better protection against oxidative damage. In addition, antioxidant enzymes such as superoxide dismutase (SOD) and catalase (CAT) showed significant improvements in T1 and T2, with T2 recording the highest activity. This confirms that clove oil supplementation strengthens the antioxidant defense system, particularly at a higher dose. Regarding inflammatory markers, tumor necrosis factor-alpha (TNF-α) levels were significantly lower in T1 and T2 (*P* = 0.003), indicating reduced inflammation. This suggests that clove oil supplementation may have anti-inflammatory properties, contributing to overall immune health. Energy reduction combined with clove oil supplementation enhanced antioxidant defense, reduced oxidative stress, and improved metabolic health. Importantly, the higher clove oil dose (T2) demonstrated stronger effects, while T1, which included an emulsifier, showed better liver function and feed efficiency. This finding indicates that clove oil, particularly at an optimized dose, support physiological balance and enhances broiler health.

**Table 4:** Effect of clove oil on blood parameters of broiler chicks.

Items	Treatment			
	Control	T1	T2	Sig
**TP (g/dl)**	4.6 ± 0.20^a^	4.03 ± 0.333^b^	4.86 ± 0.33^a^	0.008
**Alb (g/dl)**	2.133 ± 0.08^a^	1.66 ± 0.03^b^	1.73 ± 0.03^b^	0.002
**CH (mg/dl)**	145.33 ± 0.33^b^	151.66 ± 0.33^a^	154.66 ± 3.177^a^	0.031
**Creat (mg/dl)**	0.44 ± 0.02^a^	0.416 ± 0.01^a^	0.413 ± 0.006^a^	0.416
**AST (U/L)**	37.66 ± 0.33^a^	31.66 ± 0.33^b^	33.33 ± 0.33^c^	0.000
**ALT (U/L)**	9.33 ± 0.33^a^	7.5 ± 0.28^b^	9.86 ± 0.06^b^	0.002
**Antioxidant Status and Oxidative Stress Markers**				
**TAC (mmol/L**	0.75 ± 0.005^b^	1.24 ± 0.005^a^	1.17 ± 0.08^a^	0.001
**MDA (nmol/ml)**	13.7 ± 0.15^a^	8.33 ± 0.04^b^	7.03 ± 2.2^c^	0.000
**SOD (U/ml)**	46.33 ± 0.66^c^	51 ± 0.57^b^	58.33 ± 1.20^a^	0.000
**CAT (U/L)**	1.1 ± 0.05^c^	2.16 ± 0.033^b^	3.03 ± 0.033^a^	0.000
**TNF (pg/ml)**	13.66 ± 0.33^a^	12.033 ± 0.54^b^	10.13 ± 0.31^c^	0.003

Superscript letters (a, b, c) indicate significant differences within rows (*P* < 0.05).

Control: Basal diet with no supplementation, T1: Basal diet with a 200 kcal energy reduction, supplemented with 300 mg clove oil/kg diet and 1 g emulsifier/kg diet, T2: Basal diet with a 200 kcal energy reduction, supplemented with 300 mg clove oil/kg diet.

**Fig. 1:**
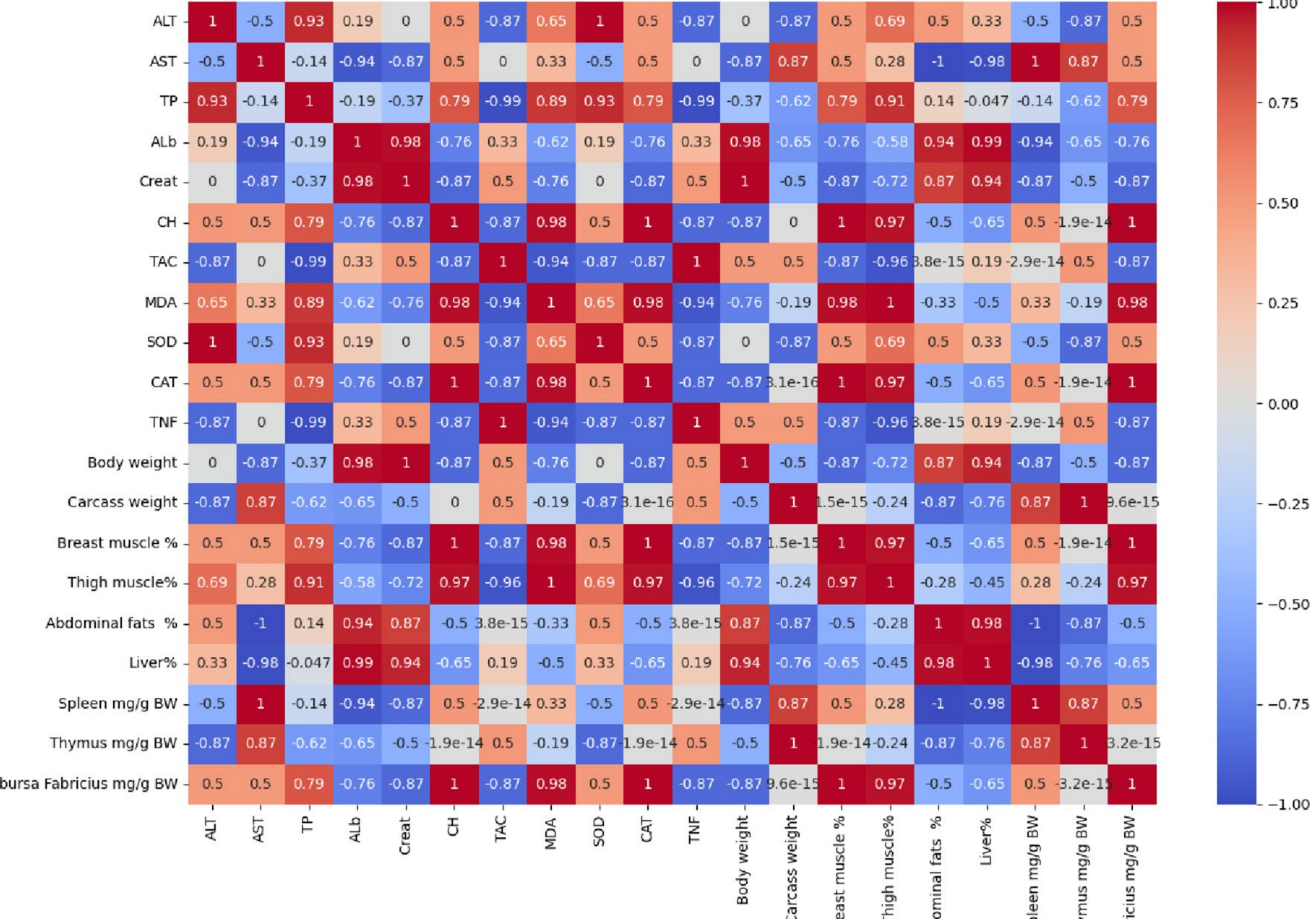
Correlation between different measured parameters in relation to control.

The results in Fig. 1 show the correlation between different measured parameters in relation to control. The results show that the total protein (TP), strongly correlated with ALb, SOD, CAT, and CH, which are represented by 0.99.0.93,0.93, and 0.73 respectively. Overall the highest antioxidant levels and total protein (Alb, TP, SOD, CAT) are strongly associated with better muscle development, higher liver weight, and lower fat deposition.

**Fig. 2:**
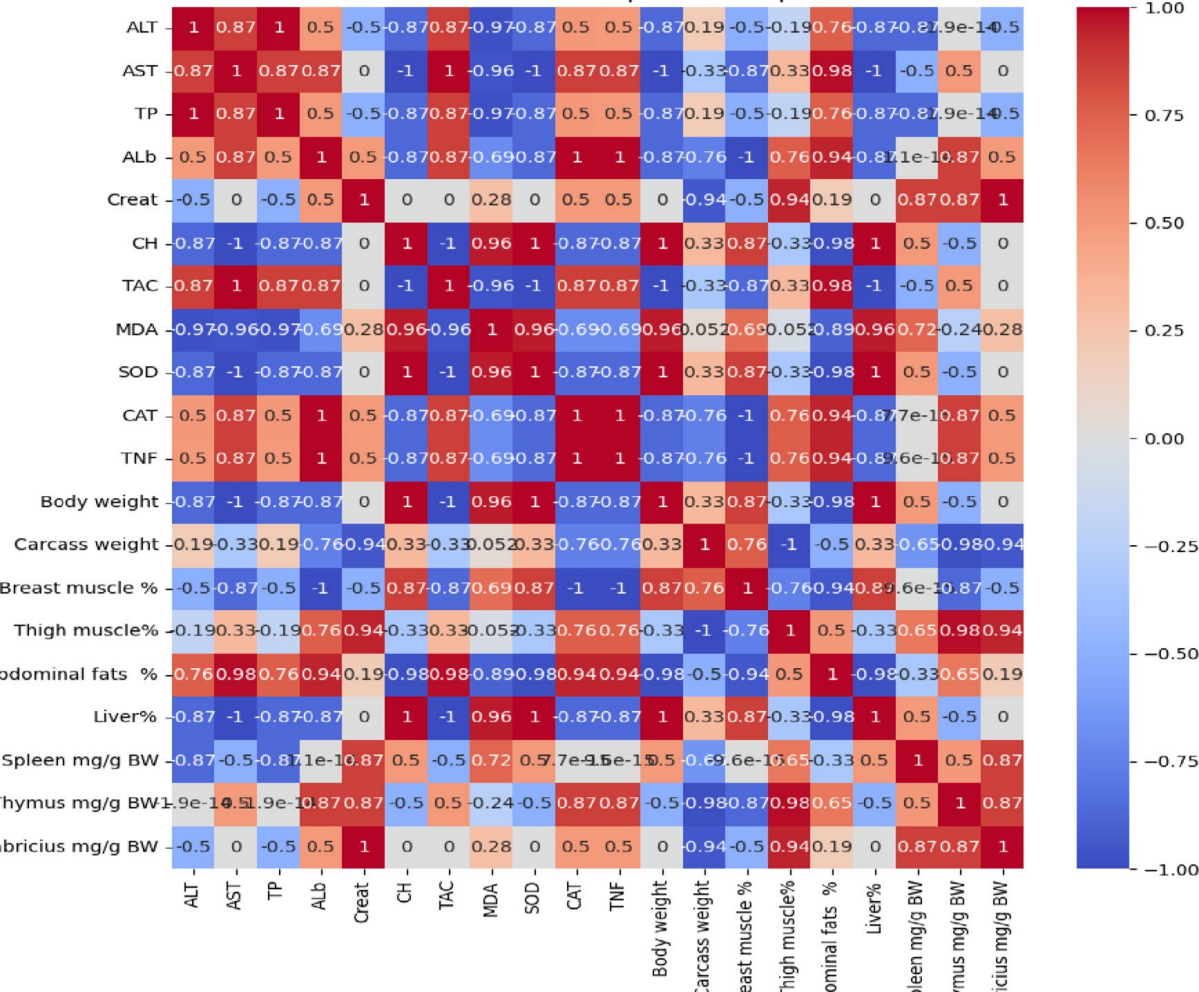
Correlation between different measured parameters in relation to treatment with clove oil with emulsifier.

The results in Fig. 2 demonstrate the heatmap correlation between different measured parameters in treatments with clove oil with emulsifier. The results demonstrate the strongly positively correlated SOD, CAT, TAC, body weight, liver%, thigh, and breastmuscle %. The higher antioxidant (TAC, SOD, CAT) and protein levels (TP, Alb) denote better body weight, liver %, and immune health.

**Fig. 3:**
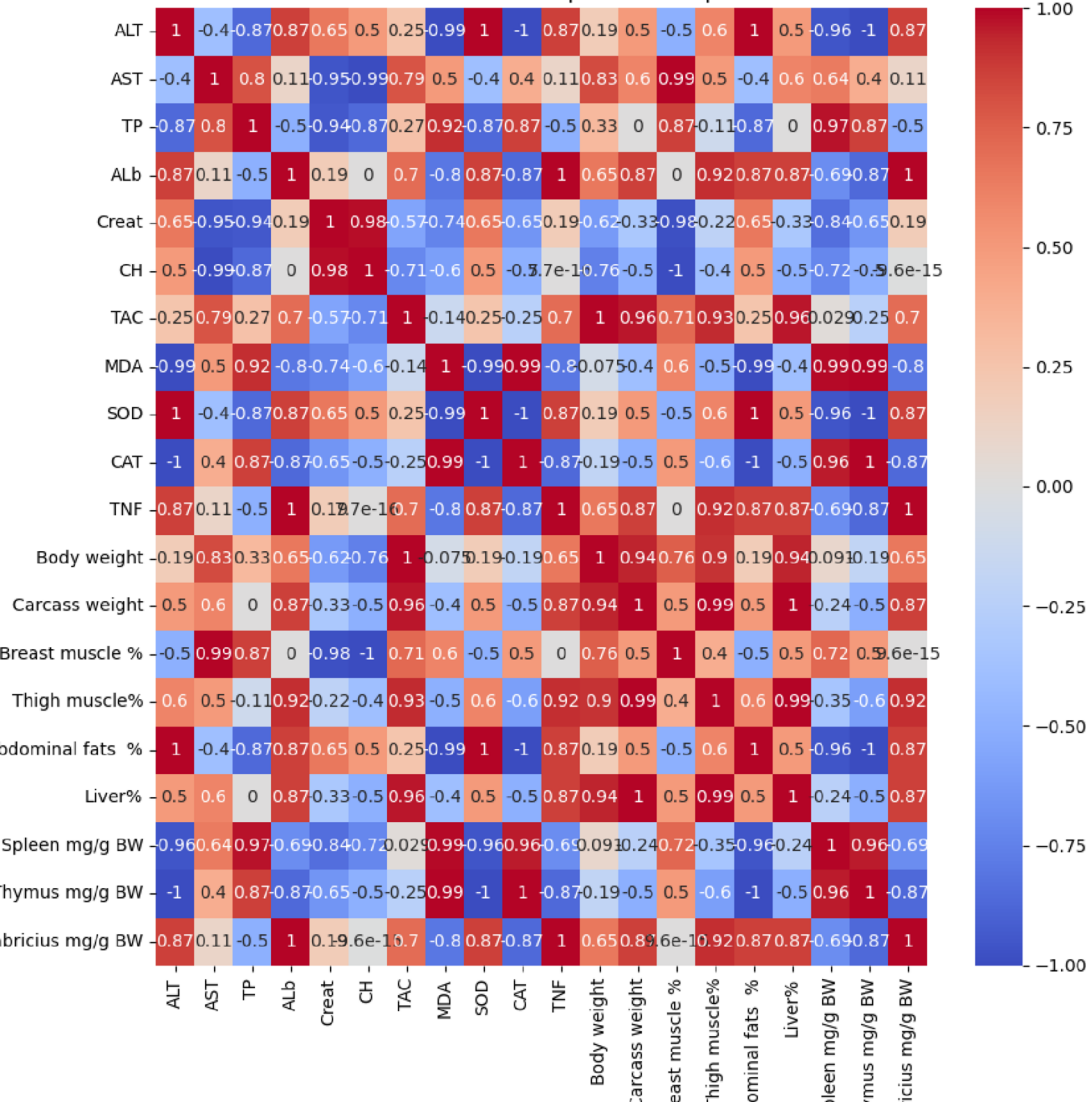
Correlation between measured parameters in relation to treatment with clove oil.

The results in Fig. 3 demonstrate the correlation between measured parameters in relation to treatment with clove oil. The results demonstrate the oxidative stress MDA strongly liver-damaged ALT and AST. The antioxidant strongly positive associated with healthy liver, muscle, protein, and immune markers. The high fat % demonstrates low antioxidant and lower muscles. The higher muscles are positively associated with Total protein (TP), Albumin (AlP), and antioxidants and negatively correlated with MDA and TNF.

### Fatty acid profile of broiler meat as influenced by emulsified clove oil, and clove oil without emulsifier supplementation

**Fig. 4:**
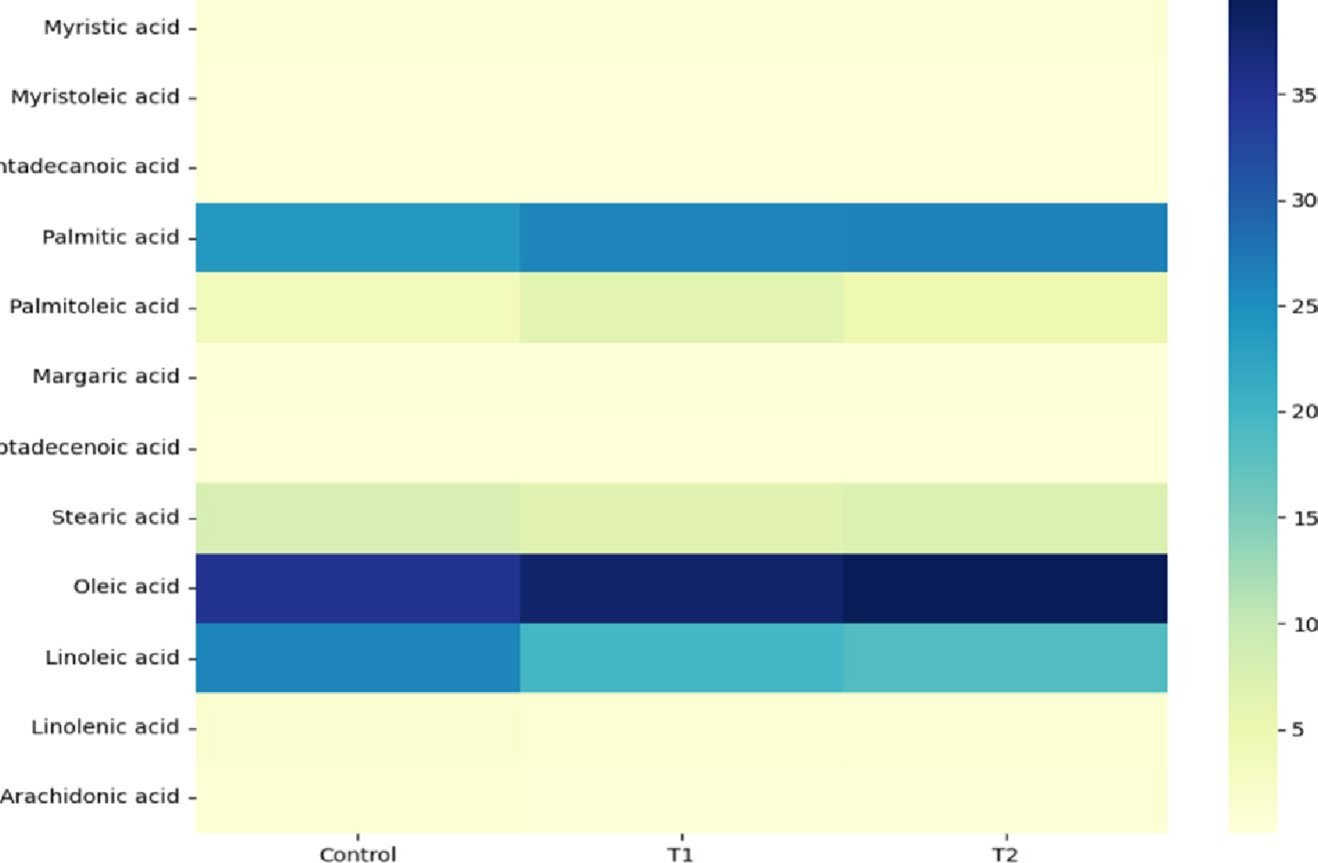
Heatmap of impact of clove oil dosage on the composition of fatty acids.

The combination of dietary energy reduction and clove oil supplementation had a clear impact on the fatty acid profile in broiler tissues (Table 5) and Fig. 4. Specifically, changes were observed in the proportions of saturated (SFA), monounsaturated (MUFA), and polyunsaturated fatty acids (PUFA). Regarding SFAs, palmitic acid increased in both T1 and T2 compared to the control, with T2 showing the highest level. Meanwhile, stearic acid slightly decreased in T1, though it remained similar between T2 and the control. These results suggest that clove oil supplementation, even with reduced dietary energy, influences the deposition of saturated fats in muscle tissues. In addition, a significant increase in oleic acid was observed in T1 and T2, with T2 exhibiting the highest level. Likewise, palmitoleic acid increased notably, particularly in T1, though it was slightly lower in T2. This indicates that clove oil supplementation, even under energy restriction, promotes the accumulation of MUFA, which is known to enhance meat quality and oxidative stability. On the other hand, PUFAs such as linoleic and linolenic acids were lower in T1 and T2 compared to the control. Moreover, arachidonic acid levels declined, particularly in T1, which could suggest a reduction in inflammatory responses. This shift implies that clove oil, when combined with reduced energy intake, helps in reducing polyunsaturated fatty acid deposition, which may contribute to better meat stability and quality.

**Table 5:** The impact of clove oil emulsifier and without emulsifier on the fatty acids composition percentage.

Rt	Compounds	Control	T1	T2
20.974	Myristic acid	0.63	0.67	0.66
22.657	Myristoleic acid	0.11	0.19	0.13
23.847	Pentadecanoic acid	0.11	0.1	0.07
26.776	Palmitic acid	23.92	26.1	26.28
27.84	Palmitoleic acid	3.7	6.48	5.17
29.429	Margaric acid	0.34	0.29	0.25
30.377	cis-10-Heptadecenoic acid	0.12	0.1	0.08
32.261	Stearic acid	7.9	6.74	7.42
33.091	Oleic acid	35	38.13	39.79
34.757	Linoleic acid	25.96	19.7	18.61
36.741	Linolenic acid	1.27	0.98	0.89
41.302	Arachidonic acid	0.94	0.51	0.65
Total		100	100	100

Control: Basal diet with no supplementation, T1: Basal diet with a 200 kcal energy reduction, supplemented with 300 mg clove oil emulsified with 1 g emulsifier/kg diet, T2: Basal diet with a 200 kcal energy and 300 mg clove oil.

### Effect of emulsified clove oil, and clove oil supplementation

**Fig. 5:**
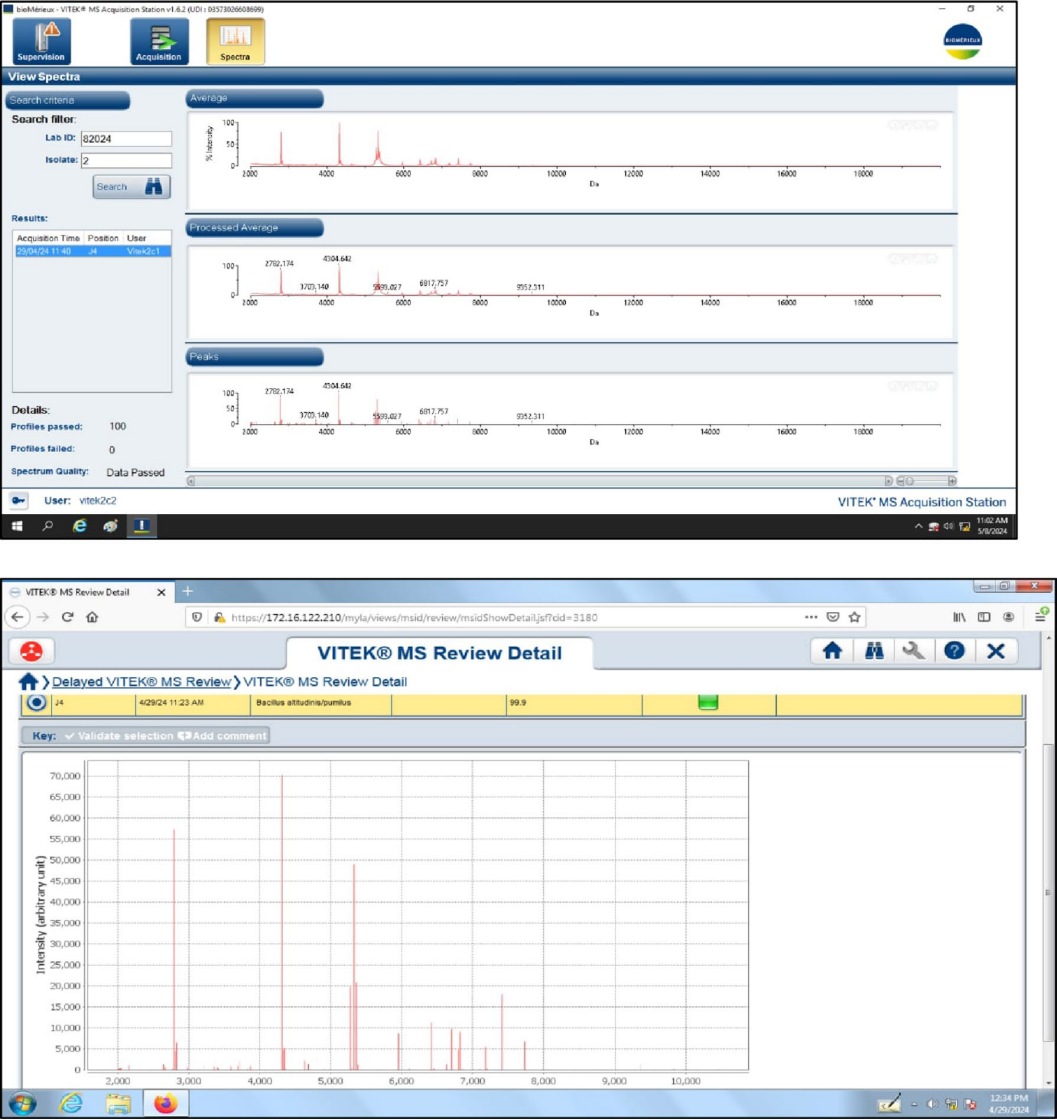
MALDI-TOF MS the spectrum of the new isolate the abdominal cavity of emulsified clove oil supplementation of broiler chicks through the reference spectra.

The average total counts were determined in Table 6. CFU·mL^− 1^ for the control, emulsified clove oil supplementation, and treatment with clove oil only respectively. Diet broilers supplemented with emulsified clove oil resulted in a significant decrease in the microbial total counts in the abdominal organs, after 35 days of breeding in comparison to control treatment. Hence, the challenged results were found after the supplementation with emulsified clove oil, where the reduction percentage is about 65% less than the control treatment. Additionally, selected colonies were purified various times and identified as *Bacillus altitudinis/pumilus* using of MALDI-TOF MS the spectrum of the new isolate showed a high match with the reference spectra, with a confidence value up to 99.9%, as shown in Fig. 5.

**Table 6:** Effect of emulsified clove oil supplementation of broiler chicks total microbial of the abdominal cavity counts.

Control	T1	T2
7.3 × 10^9^ a	3.1 × 10^4^ b	3.2 × 10^4^ b

Different letters indicates significant values.

## Discussion

### Growth performance

The results indicate that reducing dietary energy while supplementing with clove oil significantly enhanced broiler growth performance. Both treatment groups (T1: 300 mg clove oil + emulsifier, T2: 300 mg clove oil) exhibited improved BWG and FCR compared to the control, suggesting that clove oil optimizes nutrient utilization despite the energy reduction. The superior BWG observed in T2 aligns with findings by Abudabos et al.^15^, who reported that phytogenic feed additives enhance muscle deposition by stimulating digestive enzyme activity. Similarly, the improved FCR, particularly in T1, supports the conclusions of Mohiti-Asli & Ghanaatparast-Rashti^16^ that emulsifiers enhance fat digestion, leading to better feed efficiency. Interestingly, FI varied among treatments, yet the improved FCR suggests that clove oil improved feed utilization rather than increasing overall intake. This aligns with Saleh et al.^17^, who demonstrated that essential oils optimize feed efficiency by modulating gut microbiota. Moreover, the effects of clove oil became more pronounced in later growth stages, supporting the findings of Amad et al.^18^, that phytogenic additives provide cumulative benefits for digestion and metabolism as broilers age.

### Crude protein and ether extract utilization

The significant differences in crude protein utilization and ether extract utilization further confirm the efficiency of clove oil in compensating for dietary energy reduction. T1 exhibited the highest crude protein utilization, likely due to the emulsifier enhancing fat absorption and protein metabolism^19^. Similarly, both T1 and T2 showed improved ether extract utilization, demonstrating that clove oil effectively supports ether extract utilization despite lower dietary energy levels. These results align with Samal et al.^20^, who emphasized that phytogenic feed additives improve energy metabolism and nutrient absorption efficiency.

### Carcass characteristics

The higher breast and thigh muscle percentages in the supplemented groups suggest that clove oil enhances protein synthesis and lean muscle growth, even under energy-restricted conditions. The improved breast muscle yield in T2 (300 mg clove oil) indicates a dose-dependent effect on protein accretion, likely due to improved nitrogen retention and amino acid utilization^21^, Their research focuses on the role of phytogenic feed additives in improving nitrogen retention and amino acid utilization in poultry, they suggest that these additives enhance protein synthesis, which explains the improved muscle deposition observed in clove oil-supplemented groups. The presence of an emulsifier in T1 may have further optimized fat digestion and energy utilization, leading to balanced muscle deposition. These findings are consistent with those of Mohiti-Asli and Ghanaatparast-Rashti^16^, who reported that emulsifiers enhance lipid metabolism and nutrient absorption, ensuring efficient muscle development despite reduced dietary energy. Interestingly, total carcass yield remained unchanged among groups, suggesting that clove oil’s effects are more muscle-specific rather than influencing overall body composition. Moreover, the lack of significant changes in abdominal fat percentage suggests that clove oil does not contribute to excessive fat deposition, which supports findings by Fouad et al.^22^, which their work indicates that certain dietary supplements can reduce fat deposition in poultry without negatively affecting growth.

Reduced energy level significantly decreased the percentage of abdominal fat, while the introduction of emulsifier in the current study increased it. Abdominal fat can, in fact, grow with a higher rate of dietary metabolizable energy^23,24^. In a similar vein, Zaman et al.^25^, found that eating meals high in metabolizable energy increased the amount of fat in the abdomen. In comparison to low-energy diets, high-energy diets may increase the amount of body fat in broilers, which in turn may increase the amount of fat in the abdomen^26^.

The unchanged abdominal fat percentage in the current study aligns with their findings, suggesting that clove oil promotes lean muscle growth rather than fat accumulation. The observed increase in liver weight in the supplemented groups may indicate enhanced metabolic activity, potentially due to the role of essential oils in improving lipid metabolism and digestion. Additionally, the improved weight of lymphoid organs, such as the thymus and bursa Fabricius, suggests a potential immune-boosting effect of clove oil, similar to observations by **Gao**et al.^27^, they demonstrates that essential oils can stimulate immune organ development and improve disease resistance in poultry. The increased weight of lymphoid organs (thymus and bursa Fabricius) observed in the present study supports their conclusion that clove oil may enhance immune function.

### Blood biochemical parameters

The results indicate that the addition of emulsified clove oil (T1) positively influenced blood biochemical parameters, particularly under a low-energy diet. The increase in total protein (TP) in T1 suggests better protein utilization for muscle growth rather than energy production, likely due to the emulsifier’s role in enhancing fat digestion and nutrient absorption. This supports the findings of Mohiti-Asli & Ghanaatparast-Rashti^16^, who reported that emulsifiers improve fat utilization, allowing proteins to be used more efficiently for muscle development. Furthermore, the reduction in albumin (Alb) levels in T1 suggests a shift in protein usage, directing it toward tissue growth rather than circulation. This could be due to the emulsifier’s impact on nutrient distribution, ensuring optimal protein and energy use, especially under low-energy conditions. The increase in cholesterol (CH) levels in T1 suggests enhanced fat digestion and absorption, preventing excessive fat buildup in the liver. This effect is likely due to the emulsifier’s ability to facilitate fat emulsification, making fat a more efficient energy source in a low-energy diet. Saleh et al.^17^, also reported that emulsifiers improve lipid metabolism, helping broilers utilize dietary fat more effectively. Moreover, stable creatinine (Creat) levels in all groups indicate that emulsified clove oil did not negatively affect kidney function. This suggests that protein metabolism remained balanced, without placing additional stress on the kidneys. Similar findings were observed by Amad et al.^18^, who noted that phytogenic additives do not compromise renal health. A decrease in liver enzyme levels (AST and ALT), in T1 suggests that the emulsifier helped reduce metabolic stress on the liver. The antioxidant properties of clove oil may have also contributed to protecting liver cells from oxidative damage. This observation aligns with Asghari et al.^28^, who found that essential oils with emulsifiers enhance liver function by reducing oxidative stress. In contrast, the slight increase in liver enzymes in T2 (without an emulsifier) suggests a higher metabolic burden on the liver, possibly due to less efficient fat absorption. This reinforces the idea that the emulsifier not only improved fat digestion but also stabilized liver function^15^. Since poultry in low-energy diets need to maximize energy extraction from feed, the presence of an emulsifier in T1 improved fat digestion efficiency, allowing fat to be used as the primary energy source. This prevented excessive reliance on protein for energy, enabling better muscle growth and metabolic balance. These findings suggest that adding an emulsifier to clove oil improved nutrient absorption, leading to better protein and fat metabolism, improved liver function, and reduced metabolic stress in a low-energy diet. In contrast, the absence of an emulsifier in T2 resulted in a higher metabolic burden on the liver, highlighting the emulsifier’s essential role in optimizing energy use. Thus, emulsified clove oil may serve as a beneficial dietary strategy in low-energy diets, enhancing broiler performance without increasing dietary energy levels.

### Oxidative stress markers and antioxidant status

Oxidative stress refers to the imbalance between the generation and elimination of oxidants from the cells of the organism^29^. One of the primary causes of oxidative stress is the low nutritional value of poultry feeds^30^. The results indicate that emulsified clove oil supplementation improved antioxidant defense mechanisms, particularly in broilers under low-energy conditions. The increase in total antioxidant capacity (TAC) in T1 (300 mg/kg with emulsifier) suggests that the emulsifier played a key role in enhancing the bioavailability of antioxidant compounds. This is consistent with Asghari et al.^28^, who noted that plant-based bioactive compounds improve oxidative stability when efficiently absorbed. According to King et al.^31^, oxidative stabilization may be associated with phospholipids’ capacity to pool a hydrogen atom from the amino group that transports the oxidized phenolic molecule of the antioxidant. Additionally, it was clarified by Judde et al.^32^, that the antioxidant qualities of emulsifiers depend on the fatty acid structure and the quantity of copherol. The reduction in malondialdehyde (MDA) levels, particularly in T2, suggests a strong lipid peroxidation-inhibitory effect, meaning clove oil helped protect cell membranes from oxidative damage. However, T1 also showed a significant decline in MDA, indicating that the emulsifier further enhanced lipid stability and reduced oxidative stress. This supports findings by de Oliveira et al.^33^, who observed that phytogenic additives, particularly with emulsifiers, mitigate oxidative damage by stabilizing lipid metabolism. Additionally, the increased activity of superoxide dismutase (SOD) and catalase (CAT) in both treatment groups suggests that emulsified clove oil activated key enzymatic antioxidant defenses. This aligns with King et al.^31^, who found that essential oils improve oxidative stability by boosting enzymatic antioxidant activity. However, the presence of the emulsifier in T1 may have facilitated better nutrient absorption, ensuring a more balanced antioxidant response. The significant reduction in tumor necrosis factor-alpha (TNF-α) levels, particularly in T2, suggests that clove oil exerts anti-inflammatory effects. However, the moderate reduction in TNF-α in T1 indicates that the emulsifier contributed to a more controlled inflammatory response, preventing excessive immune activation. This observation supports Saleh et al.^17^, who reported that phytogenic compounds regulate immune responses and reduce systemic inflammation. Furthermore, bioactive phenolic compounds in clove oil may have contributed to immunomodulation by regulating inflammatory cytokines^17^. This is particularly relevant in low-energy diets, where oxidative and inflammatory stress may be higher due to metabolic adjustments. Although both 300 mg/kg (T1) and 300 mg/kg (T2) doses significantly improved antioxidant status and reduced oxidative stress, T1 appeared to maintain a more stable metabolic response, particularly in liver enzyme activity. This suggests that moderate levels (300 mg/kg) may provide optimal antioxidant and immune benefits without potential metabolic fluctuations associated with higher doses^32^. The findings suggest that emulsified clove oil enhances antioxidant defenses, reduces lipid peroxidation, and supports an anti-inflammatory response, particularly in low-energy diets. The emulsifier in T1 likely improved nutrient absorption and bioavailability, ensuring efficient utilization of antioxidant compounds. While higher doses (T2) provided stronger oxidative stress protection, moderate doses (T1) ensured metabolic stability, highlighting the emulsifier’s role in optimizing the benefits of clove oil supplementation.

### Facids composition of

The results of this study indicate that emulsified clove oil supplementation significantly influenced the fatty acid profile of broiler meat, particularly under a low-energy diet. This suggests that clove oil, especially when combined with an emulsifier (T1), can enhance fat digestion and utilization, thereby modifying lipid metabolism. Notably, the observed increase in saturated fatty acids (SFA), particularly palmitic and myristic acids, suggests that clove oil supplementation alters fat deposition. To increase the digestion of saturated fatty acids, emulsifiers can help dissolve free fatty acids that are rarely soluble on their own in bile salt micelles^17^.

Additionally, the slight reduction in stearic acid in T1, but its maintenance at control levels in T2, suggests that the emulsifier in T1 may have contributed to a more efficient fat metabolism by improving lipid emulsification and absorption. Moreover, the significant increase in monounsaturated fatty acids (MUFA), particularly oleic acid, in both treatment groups reinforces previous studies highlighting the beneficial effects of phytogenic feed additives on lipid metabolism^34^. However, the highest oleic acid accumulation in T2 suggests a dose-dependent effect of clove oil, while T1’s balanced MUFA levels indicate that the emulsifier may have enhanced lipid utilization without excessive fat deposition. Given that oleic acid is associated with improved meat quality and oxidative stability^36^, its increase in clove oil-supplemented groups suggests a positive modification in broiler meat composition. Furthermore, the sharp rise in palmitoleic acid, particularly in T1, supports the hypothesis that emulsifiers facilitate the absorption and transport of MUFAs, which are essential for maintaining lipid balance and meat palatability^34^. On the other hand, the reduction in polyunsaturated fatty acids (PUFA), including linoleic and linolenic acids, suggests that clove oil may contribute to stabilizing meat lipids by reducing susceptibility to oxidation. This finding aligns with Saleh et al.^17^, who reported that essential oils exhibit antioxidant properties that influence fatty acid composition. Likewise, the decrease in arachidonic acid further supports the hypothesis that clove oil supplementation reduces PUFA levels, potentially minimizing lipid peroxidation and improving oxidative stability. Since high PUFA content can make meat more prone to rancidity Amad et al.^18^, the observed reduction in linoleic and linolenic acids in both T1 and T2 suggests a protective role of clove oil in preserving meat quality. Additionally, the emulsifier in T1 may have played a role in stabilizing fat metabolism, preventing excessive lipid oxidation, as noted by Roy et al.,_36_ Furthermore, the minor changes observed in other fatty acids, such as margaric acid, pentadecanoic acid, and cis-10-heptadecenoic acid, indicate that clove oil primarily affects major fatty acid groups rather than minor components. This selective modification suggests a targeted metabolic effect rather than a broad alteration in lipid composition^35,36^. Overall, these findings suggest that clove oil supplementation at both emulsifier 300 mg/kg (T1) and 300 mg/kg (T2) without emulsifier enhances the fatty acid profile of broiler meat, even under a low-energy diet. However, while T2 exhibited the most pronounced effects, T1, with the addition of an emulsifier, demonstrated a more balanced fatty acid composition, indicating improved lipid utilization. This suggests that in energy-restricted conditions, emulsifiers may play a crucial role in optimizing fat digestion and absorption. Consequently, further research is needed to explore the long-term implications of these fatty acid modifications on meat storage, sensory attributes, and consumer preferences.

Clove essential oil, rich in bioactive components like eugenol, can enhance growth performance, immune status, and digestive health in broiler chickens by promoting a healthier gut environment, enhancing nutrient absorption, and boosting immunity^4^. Clove extracts’ mode of action could be influenced by changes in the gut microbiota and epithelium^37^.

## Conclusion

This study demonstrated that emulsified clove oil supplementation positively influenced broiler performance, carcass traits, blood biochemistry, antioxidant status, and meat quality under a low-energy diet. The inclusion of an emulsifier improved nutrient absorption and feed efficiency, supporting balanced growth. While the higher dose enhanced muscle deposition and antioxidant activity, the lower dose with an emulsifier provided more stable metabolic responses. Additionally, clove oil improved lipid metabolism and oxidative stability, contributing to better meat quality. These findings suggest that emulsified clove oil is an effective feed additive for optimizing broiler production in energy-restricted diets.

## Data Availability

The datasets spent and/or analyzed during this study are available from the corresponding author upon reason able request.

## References

[1] Naser, K. M. B., Sherif, B. M., Othman, S. M. & Asheg, A. A. Effect of clove buds powder supplementation on hematological profile, biochemical parameters, lymphoid organs, and cell-mediated immunity of broilers. *Open. Veterinary J.* **13** (7), 854–863. 10.5455/OVJ.2023.v13.i7.7 (2023). 10.5455/OVJ.2023.v13.i7.7 PMC10443825 37614736

[2] Brown, K., Uwiera, R. R., Kalmokoff, M. L., Brooks, S. P. & Inglis, G. D. Antimicrobial growth promoter use in livestock: a requirement to understand their modes of action to develop effective alternatives. *Int. J. Antimicrob. Agents*. **49** (1), 12–24 (2017). 27717740 10.1016/j.ijantimicag.2016.08.006

[3] Wang, J. et al. Phytogenic feed additives as natural antibiotic alternatives in animal health and production: A review of the literature of the last decade. *Anim. Nutr.*10.1016/j.aninu.2024.01.012 (2024). 38800730 10.1016/j.aninu.2024.01.012 PMC11127233

[4] Elbaz, A. M. et al. Effect of different levels of clove essential oil on the growth performance, lipid metabolism, immunity, and intestinal microbial structure of broiler chickens. *Egypt. J. Nutr. Feeds*. **25** (3), 361–368. 10.21608/ejnf.2022.286670 (2022).

[5] Karaca, N., Demirci, B., Gavahian, M. & Demirci, F. Enhanced bioactivity of rosemary, sage, lavender, and chamomile essential oils by fractionation, combination, and emulsification. *ACS Omega*. **8** (12), 10941–10953. 10.1021/acsomega.2c07508 (2023). 37008100 10.1021/acsomega.2c07508 PMC10061596

[6] Hashemipour, H., Kermanshahi, H., Golian, A. & Veldkamp, T. J. P. S. Effect of thymol and carvacrol feed supplementation on performance, antioxidant enzyme activities, fatty acid composition, digestive enzyme activities, and immune response in broiler chickens. *Poult. Sci.* **92** (8), 2059–2069. 10.3382/ps.2012-02685 (2013). 23873553 10.3382/ps.2012-02685

[7] Shahbazi, Y. Antioxidant, antibacterial, and antifungal properties of nanoemulsion of clove essential oil. Nanomedicine Research Journal. Dec 1;4(4):204-8 (2019).

[8] Wang, W., Zhang, Y., Yang, Z. & He, Q. Effects of incorporation with clove (*Eugenia caryophyllata*) essential oil (CEO) on overall performance of Chitosan as active coating. *Int. J. Biol. Macromol.* **166**, 578–586 (2021). 33137383 10.1016/j.ijbiomac.2020.10.215

[9] Elbaz, A. M. et al. Effectiveness of probiotics and clove essential oils in improving growth performance, immuno-antioxidant status, ileum morphometric, and microbial community structure for heat-stressed broilers. *Scientific Reports*, *13*(1), p.18846. (2023). 10.1038/s41598-023-45868-9 PMC10620235 37914748

[10] Henao-Ardila, A., Quintanilla-Carvajal, M. X. & Moreno, F. L. Emulsification and stabilization technologies used for the inclusion of lipophilic functional ingredients in food systems. *Heliyon*10.1016/j.heliyon.2024.e32150 (2024). 38873677 10.1016/j.heliyon.2024.e32150 PMC11170136

[11] NRC. *National Research Council, & Subcommittee on Poultry Nutrition. Nutrient Requirements of Poultry: 1994* (National Academies, 1994).

[12] Avino, P., Campanella, L. & Russo, M. V. High-performance liquid chromatography intercomparative study for amino acid analysis in two tissues by PITC-and OPA-derivatizations. *Anal. Lett.* **34** (6), 867–882 (2001).

[13] Mitsch, P. et al. The effect of two different blends of essential oil components on the proliferation of clostridium perfringens in the intestines of broiler chickens. *Poult. Sci.* **83** (4), 669–675. 10.1093/ps/83.4.669 (2004). 15109065 10.1093/ps/83.4.669

[14] Duncan, D. B. Multiple range and multiple F tests. *Biometrics* **11** (1), 1–42 (1955).

[15] Abudabos, A. M., Alyemni, A. H., Dafalla, Y. M. & Khan, R. U. The effect of phytogenics on growth traits, blood biochemical and intestinal histology in broiler chickens exposed to *Clostridium perfringens* challenge. *J. Appl. Anim. Res.* **46** (1), 691–695 (2018).

[16] Mohiti-Asli, M. & Ghanaatparast-Rashti, M. Dietary oregano essential oil alleviates experimentally induced coccidiosis in broilers. *Prev. Vet. Med.* **120** (2), 195–202. 10.1016/j.prevetmed.03.014 (2015). 25864115 10.1016/j.prevetmed.2015.03.014

[17] Saleh, A. A., Amber, K. A., Mousa, M. M., Nada, A. L., Awad, W., Dawood, M. A., …Abdel-Daim, M. M. A mixture of exogenous emulsifiers increased the acceptance of broilers to low energy diets: Growth performance, blood chemistry, and fatty acids traits.Animals, 10(3), 437. (2020). doi:10.3390/ani10030437. 10.3390/ani10030437 PMC7142428 32150863

[18] Amad, A. A., Wendler, K. R. & Zentek, J. Effects of a phytogenic feed additive on growth performance, selected blood criteria and jejunal morphology in broiler chickens. *Emirates J. Food Agric. (EJFA)*. **25** (7). 10.9755/ejfa.v25i7.12364 (2013).

[19] Chowdhury, S., Mandal, G. P. & Patra, A. K. Different essential oils in diets of chickens: 1. Growth performance, nutrient utilisation, nitrogen excretion, carcass traits and chemical composition of meat. *Anim. Feed Sci. Technol.* **236**, 86–97. 10.1016/j.anifeedsci.2017.12.002 (2018).

[20] Samal, L., Chaudhary, L. C., Agarwal, N. & Kamra, D. N. Impact of phytogenic feed additives on growth performance, nutrient digestion and methanogenesis in growing buffaloes. *Anim. Prod. Sci.* **58** (6), 1056–1063 (2016).

[21] Abdelli, N., Solà-Oriol, D. & Pérez, J. F. Phytogenic feed additives in poultry: achievements, prospective and challenges. *Animals* **11** (12), 3471. 10.3390/ani11123471 (2021). 34944248 10.3390/ani11123471 PMC8698016

[22] Fouad, A. M. & El-Senousey, H. K. Nutritional factors affecting abdominal fat deposition in poultry: a review. *Asian-Australasian J. Anim. Sci.* **27** (7), 1057. 10.5713/ajas.2013.13702 (2014). 10.5713/ajas.2013.13702 PMC4093572 25050050

[23] Vahdatpour, T., Nazer-Adl, K., Ebrahim-Nezhad, Y., Maheri-Sis, N. & Vahdatpour, S. The effects of energy increasing and protein Lowering by addition of fats to diet on broiler chickens: performance and serum lipids. *Asian J. Anim. Veterinary Adv.* **3** (5), 286–292 (2008).

[24] Saleh, A. A. et al. Synergistic effect of feeding Aspergillus Awamori and lactic acid bacteria on performance, egg traits, egg yolk cholesterol and fatty acid profile in laying hens. *Italian J. Anim. Sci.* **16** (1), 132–139 (2017).

[25] Zaman, Q. U. et al. Effect of varying dietary energy and protein on broiler performance in hot climate. *Anim. Feed Sci. Technol.* **146** (3–4), 302–312 (2008).

[26] Nahashon, S. N., Adefope, N., Amenyenu, A. & Wright, D. Effects of dietary metabolizable energy and crude protein concentrations on growth performance and carcass characteristics of French Guinea broilers. *Poult. Sci.* **84** (2), 337–344 (2005). 15742972 10.1093/ps/84.2.337

[27] Gao, J. et al. Effect of plant essential oil on growth performance and immune function during rearing period in laying hens. *Brazilian J. Poult. Sci.* **22** (03), eRBCA–2019. 10.1590/1806-9061-2019-1244 (2020).

[28] Asghari, M. et al. Effects of emulsified essential oils blend on performance, blood metabolites, oxidative status and intestinal microflora of suckling calves. *Animal Feed Science and Technology*, *277*, p.114954. (2021).

[29] El-Deep, M. H., Dawood, M. A. O., Assar, M. H., Ijiri, D. & Ohtsuka, A. Dietary Moringa Oleifera improves growth performance, oxidative status, and immune related gene expression in broilers under normal and high temperature conditions. *J. Therm. Biol*. **82**, 157–163 (2019). 31128643 10.1016/j.jtherbio.2019.04.016

[30] Zangeneh, S., Torki, M., Lotfollahian, H. & Abdolmohammadi, A. Effects of dietary supplemental lysophospholipids and vitamin C on performance, antioxidant enzymes, lipid peroxidation, thyroid hormones and serum metabolites of broiler chickens reared under thermoneutral and high ambient temperature. *J. Anim. Physiol. Anim. Nutr.* **102** (6), 1521–1532 (2018). 10.1111/jpn.12935 30255521

[31] King, M. F., Boyd, L. C. & Sheldon, B. W. Antioxidant properties of individual phospholipids in a salmon oil model system. *J. Am. Oil Chem. Soc.* **69**, 545–551 (1992).

[32] Judde, A., Villeneuve, P., Rossignol-Castera, A. & Le Guillou, A. Antioxidant effect of soy lecithins on vegetable oil stability and their synergism with tocopherols. *J. Am. Oil Chem. Soc.* **80** (12), 1209–1215 (2003).

[33] de Oliveira, L. S., Balbino, E. M., Silva, T. N. S., Ily, L., da Rocha, T. C., Strada,E. S. D. O., … de Brito, J. Á. G. Use of emulsifier and lipase in feeds for broiler chickens,40, (6), 3181–3196, (2019).

[34] Zhang, B., Haitao, L., Zhao, D., Guo, Y. & Barri, A. Effect of fat type and lysophosphatidylcholine addition to broiler diets on performance, apparent digestibility of fatty acids, and apparent metabolizable energy content. *Anim. Feed Sci. Technol.* **163** (2–4), 177–184 (2011).

[35] Wang, J. P., Zhang, Z. F., Yan, L. & Kim, I. H. Effects of dietary supplementation of emulsifier and carbohydrase on the growth performance, serum cholesterol and breast meat fatty acids profile of broiler chickens. *Anim. Sci. J.* **87** (2), 250–256. 10.1111/asj.12412 (2016). 26278708 10.1111/asj.12412

[36] Roy, A., Haldar, S., Mondal, S. & Ghosh, T. K. Effects of supplemental exogenous emulsifier on performance, nutrient metabolism, and serum lipid profile in broiler chickens. Veterinary medicine international, 262604 (2010). (2010) (1). 10.4061/2010/262604 PMC2910456 20671938

[37] Agostini, P. S. et al. Role of in-feed clove supplementation on growth performance, intestinal microbiology, and morphology in broiler chicken. *Livest. Sci.* **147** (1–3), 113–118. 10.1016/j.livsci.2012.04.010 (2012).

